# Caregiver burden and its determinants among family members of patients with chronic viral hepatitis in Shanghai, China: a community-based survey

**DOI:** 10.1186/1471-2334-14-82

**Published:** 2014-02-12

**Authors:** Hong Ren, Yan Yu, Jia-Yu Hu, Yang Shi, Yi-Han Lu, Wei Meng

**Affiliations:** 1 Department of Epidemiology, The Key Laboratory of Public Health Safety of Minister of Education, Fudan University School of Public Health, Building 8 Room 429,130 Dong’an Road, Shanghai 200032, China; 2 Department of Infectious Disease Control and Prevention, Shanghai Municipal Center for Disease Control and Prevention, Shanghai 200336, China; 3 Department of Injury Control and Prevention, Shanghai Municipal Center for Disease Control and Prevention, Shanghai 200336, China

**Keywords:** Chronic viral hepatitis, Family caregiver, The family burden interview schedule, Reliability and validity

## Abstract

**Background:**

In China, caregivers of chronic viral hepatitis patients experience considerable burdens, stress and disruption of their own well-being and social activities. Measurement of the effect on caregivers is an under-researched area. The Family Burden Interview Schedule (FBIS) was primarily devised for the caregivers of schizophrenia patients, and the adverse effect of the disease was similar to the effect of chronic viral hepatitis on family caregivers. In this study, we prospectively evaluated the psychometric properties of FBIS in the field of chronic viral hepatitis and used it to determine the factors affecting the caregiver burden on the family members of chronic viral hepatitis patients in Shanghai, China.

**Methods:**

A representative sample of patients (n = 1478) and caregivers (n = 1478) was randomly obtained through a multi-stage cluster sampling in Shanghai, China. Reliability and validity tests were used to verify the psychometric properties of the instrument. The two-level random intercept model was applied to determine the factors of the caregiver burden between the household and the community level.

**Results:**

Cronbach’s alpha coefficient was 0.90 for the overall instrument with statistical significance. Factor analysis suggested a three-factor model for the FBIS and confirmed that the adjusted unidimensional model and the second-order multidimensional model had better fit statistics. The average score of the caregiver burden in Shanghai was 12.62 ± 10.74, and financial burden constituted the major effect. The two-level random intercept model demonstrated that the risk factors were hospitalisation (β 1.69, 95%CI 0.48 to 2.90), elevated serum alanine aminotransferase levels (β 1.05, 95%CI 0.15 to 1.95), HCV infection (β 4.49, 95%CI 1.22 to 7.77), and acceptance of the hepatitis B vaccine (β 2.20, 95%CI 0.56 to 3.85), whereas the protective factors were no consumption of alcohol (β -2.69, 95%CI −5.19 to −0.19), average monthly costs for patients less than or equal to 100 US dollars (β -2.96, 95%CI −5.83 to −0.09), and good health status of family caregivers (β -9.91, 95%CI −12.76 to −7.05).

**Conclusions:**

FBIS can accurately measure the caregiver burden for chronic hepatitis. Targeting interventions toward the conditions associated with the caregiver burden is of great importance.

## Background

Hepatitis B virus (HBV) and hepatitis C virus (HCV) infections are the leading causes of chronic hepatitis, cirrhosis, and hepatocellular carcinoma (HCC) worldwide, especially in the Asia-Pacific region [1-3]. The prevalence of HBV in China is one of the highest in the world, and nearly 10% of the population has HBV; the nationwide prevalence of HCV infection in 1992 was estimated to be 3.2% [4]. The disease burden of HBV infection and HCC in China is hypothesised to be among the largest in the world, and that of HCV infection is likely to be substantial as well [4]. In past decades, measures against HBV infections in China primarily depended on neonatal immunisation [5], and those against HCV infection were restricted to the prevention of high risk factors [6]. Thus, understanding the caregiver burdens and the needs of patients with chronic viral hepatitis is necessary.

The traditional biomedical model of health is being integrated with the social science model of health, which is based on a psychosocial and economic foundation. This integrated approach to clinical practice and research on hepatitis prevention requires monitoring the traditional physiological and biomedical outcomes and improving the community environment, family support, and health related quality of life [7,8]. In China, family members in the role of caregiver of patients with chronic viral hepatitis experience considerable burdens, stress, disruption of personal wellbeing and social activities. In most cases, they worry about the economic effect, therapeutic effect and time expenditure, and are at risk for emotional and physical health problems. Measurement of the family caregiver burden of chronic hepatitis patients using an appropriate psychometric instrument has been an important yet under-researched area in the Chinese population.

The Family Burden Interview Schedule (FBIS) was primarily devised for the family caregivers for schizophrenia patients by Pai and Kapur in India in 1981 [9]. The instrument has been used in a number of studies to evaluate with satisfactory psychometric properties the caregiver burden among families of neurotic patients [10], patients with alcohol and opioid dependence [11], and patients with intellectual disability [12]. In 2004, the original English version of FBIS was translated into Chinese and used to measure the caregiver burden among Chinese patients with schizophrenia [13], traumatic brain injury [14], and schistosomiasis [15]. The characteristics of chronic viral hepatitis patients and the adverse effect on their family caregivers are complex and similar to those of other chronic diseases. In this study, we aimed to conduct a community-based survey to examine the reliability and validity of FBIS in chronic viral hepatitis cases and to use it to determine the burden and factors reported by caregivers of chronic viral hepatitis patients in Shanghai, China.

## Methods

### Data source and sampling method

Chronic viral hepatitis is a nationally notifiable infectious disease in China. Upon laboratory confirmation, hospital physicians must register each patient’s information in the national infectious disease online report system within 24 hours. Community physicians then conduct an epidemiological investigation, health education, and long-time follow up of each patient and their family members. Shanghai is one of the largest metropolitan areas in China, and approximately 30,000 chronic viral hepatitis patients have been followed up in Shanghai. We directly recruited patients and their family caregivers for this study from communities through a three-stage cluster sampling of households.

Shanghai is administratively divided into 16 districts and 1 county. In this study, 5 urban districts, 4 suburban districts, and 1 county were firstly randomly selected as the study districts. Then four communities were selected from each district as the study communities. In a total of 40 communities, every registered chronic viral hepatitis patient and one family caregiver of each patient were enrolled as the target population.

### Inclusion criteria

Patients of either sex in the age range 18 to 70 years were included in our study if they met the following criteria: 1) Diagnosed at municipal hospitals in Shanghai according to the *Guideline on the Prevention and Treatment of Chronic Hepatitis B in China (2005)*[16], the *Diagnosis, Management, and Treatment of Hepatitis C: an Update*[17], and the *Guideline of Prevention and Treatment of Hepatitis C*[18]; 2) Locally registered and living in the study community during 2012; and 3) Without mental illness, personality disorders, organic brain disease or substance abuse.

Family caregivers were included when they satisfied the following criteria: 1) The principal caregiver of the patient; 2) Healthy adults aged 18 years or more; 3) Staying with the patient currently and for at least three previous years; 4) No history of HBV and HCV infections; 5) No history of mental illness, personality disorder or substance abuse according to The Chinese Classification of Mental Disorders version 3 (CCMD-III).

### Epidemiological investigation and instrument

Chronic viral hepatitis patients and a family caregiver of each patient were interviewed separately by trained research assistants using different questionnaires requesting information up to 6 months before the investigation. The patients’ information included demographics, health status, diagnosis and therapy (medical records), cognition and behaviour, acceptance of public health service, and socioeconomic status. The family caregivers’ information included demographics, health status, immunisation history, the average monthly patient costs, and the family burden determined by FBIS.

FBIS is a semi-structured interview instrument composed of 25 items that are grouped into the following 6 scales (Table 1): financial burden (items 1–8), disruption of family routine activities (items 7–11), disruption of family leisure (items 12–15), disruption of family interactions (items 16–20), the effect on the physical health of others (items 21–22), and the effect on the mental health of others (items 23–25). Each item was rated on a three-point scale, where 0 was no burden, 1 was a moderate burden, and 2 was a severe burden. The total scores ranged from 0 to 50 with 50 indicating the highest burden of care [11].

**Table 1:** Factor loadings of the three-factor solution for FBIS (n = 739)

**Scales**	**Items**	**Factor 1**	**Factor 2**	**Factor 3**
		**Eigenval**	**Eigenval**	**Eigenval**
		**ue = 13.80**	**ue = 2.31**	**ue = 1.43**
Financial burden	1. Loss of patient’s income		0.84*	
	2. Loss of income of other family members		0.78*	
	3. Expenses of patient’s illness		0.81*	
	4. Expenses due to other necessary changes in arrangements		0.57	
	5. Loans taken		0.78*	
	6. Any other planned activity needing finance, postponed		0.71	
Disruption of family routine activities	7. Patient not attending work, school, etc.		0.70	
	8. Patient unable to help in household duties	0.52	0.58	
	9. Disruption of activities due to patient’s illness and care	0.62		
	10. Disruption of activities due to patient’s irrational demands	0.68		
	11. Other family members missing school, meals, etc.	0.69		
Disruption of family leisure	12. Stopping of normal recreational activities	0.77*		
	13. Absorption of another member’s holiday and leisure time	0.76*		
	14. Lack of participation by patient in leisure activity	0.80*		
	15. Planned leisure activity abandoned	0.80*		
Disruption of family interactions	16. Effect on general family atmosphere	0.58		
	17. Other members arguing over the patient	0.55		0.55
	18. Reduction or cessation of interaction with friends and neighbors	0.68		
	19. Family becoming secluded or withdrawn	0.71*		
	20. Any other effect on family or neighborhood relationships	0.68		
Effect on physical health of others	21. Physical illness in any family member			0.78*
	22. Any other adverse effect on others			0.78*
Effect on mental health of others	23. Any member seeking professional help for psychological illness			0.82*
	24. Any member becoming depressed, weepy, irritable			0.84*
	25. How you have suffered owing to patient’s illness			0.84*

Factor loadings more than or equal to 0.70 have been marked with an asterisk (*). Factor loadings smaller than 0.50 have not been presented.

### Data processing and analysis

The data were established using the free software EpiData Association version 3.1 (http://www.epidata.dk/download.php) and cross-checked with double entry. The descriptive statistics, split half reliability, internal consistency reliability, convergent and discriminative validity, exploratory factor analysis, and two-level random intercept model were determined using SPSS for Windows version 17.0 (Chicago, IL, USA). The confirmatory factor analysis of FBIS was conducted by SPSS Amos version 17.0.

The reliability analyses included split half reliability and internal consistency reliability. The split half reliability was measured with the Spearman-Brown Prediction formula. The internal consistency reliability was measured by Cronbach’s alpha coefficient (≥ 0.70 was considered as evidence of acceptable reliability) [19].

The validity analyses included convergent, discriminant and structural validity. The convergent validity was examined using the hypothesised item-scale correlation. The discriminant validity was assessed using the correlation of an item with other scales. A correlation of ≥ 0.40 was considered as a better convergent validity, whereas the discriminant validity was supported whenever the hypothesised item-scale correlation was higher than the alternative ones [20,21]. To examine the structural validity of FBIS, the variable “grouping” was generated through the method of using a random number in the database. The database was evenly divided into odd and even halves from the newly generated variable for the exploratory and confirmatory factor analysis, respectively. The exploratory factor analysis was explored using principal component factor analysis with varimax rotation with Kaiser Normalization, and the factors were extracted with an eigenvalue ≥ 1.0 [22]. The confirmatory factor analysis was conducted using structural equation models to assess the performance and structure of FBIS with the Goodness-of-Fit Index (GFI), the Adjusted Goodness-of-Fit Index (AGFI), the Normed Fit Index (NFI), and the Comparative Fit Index (CFI) of >0.90, as well as the Root Mean Square Residuals (RMR) of <0.05 and the Root Mean Square Error of Approximation (RMSER) of <0.08 [23].

Considering the hierarchical structure of the database, the two-level random intercept model was applied to identify the independent variables of the family caregiver burden in chronic viral hepatitis cases on the household and the community levels [24]. The outcome was determined with the use of factors including the demographics, health status, diagnosis, cognition and behaviour, acceptance of public health service, and economic costs of patients or their family caregivers. A *P* value of <0.05 was considered to indicate statistical significance.

### Ethics statement

The study protocol was reviewed and approved by the Human Research Ethics Committee of Shanghai Municipal Center for Disease Control and Prevention. Written informed consent was obtained from each recruited patient and family caregiver before the questionnaire survey.

## Results

### Demographics

Excluding those who refused participation (20) and/or had missing data (2), 1478 chronic viral hepatitis patients and one family caregiver of each patient were recruited from 10 districts and 40 communities in Shanghai during 2012. Males accounted for 64.34% (951/1478) of the patients. The mean age of the males in the study was 50.29 ± 12.95 years, and that of females was 52.70 ± 13.74 years. In the top three ranking occupations of the patients, 505 (34.17%) were retirees, 301 (20.37%) were workers and 216 (14.61%) were householders. Of the patients, 259 (17.52%) graduated from university, 460 (31.12%) from high school, 561 (37.96%) from middle school, and 137 (9.27%) from elementary school; 61 (4.13%) had no formal education.

Of the family caregivers interviewed, females accounted for 62.04% (917/1478). The mean age of the males was 53.35 ± 14.39 years and that of the females was 50.05 ± 13.04 years. Considering the relationship between the patients and family caregivers, 139 (9.40%) were parents, 1133 (76.66%) were a spouse, and 206 (13.94%) were siblings or children. The mean nursing time was 12.95 ± 12.31 years (spouse), 13.00 ± 13.73 years (parents), and 9.85 ± 11.57 years (siblings or children).

### Reliability and validity of FBIS for chronic viral hepatitis

Split half reliability was used to compare the similarities of the items separated into odd and even halves. In our study, the Pearson correlation coefficient was 0.96 with statistical significance (*P* < 0.001), and the split half reliability with Spearman-Brown Prediction formula was 0.98.

Cronbach’s alpha coefficient for the FBIS was 0.90 for the overall instrument and 0.88 for “financial burden”, 0.89 for “disruption of family routine activities”, 0.89 for “disruption of family leisure”, 0.88 for “disruption of family interactions”, 0.91 for “effect on physical health of others”, and 0.90 for “effect on mental health of others” with statistical significance (*P* < 0.001).

The convergent and discriminant validity of the FBIS is listed in Table 2, which shows all the items that met the criterion for item-convergent validity (item-scale correlations ≥ 0.40), and 118 of the 125 hypothesised item-scale correlations were higher than the alternative ones, except for item 4 and 7. The correlation of item 4 (i.e., expenses due to other necessary changes in arrangements) with the “financial burden” scale had no significant difference compared with the alternative scale of “disruption of family routine activities” (*t* = 1.68, *P* > 0.05). The correlation of item 7 (i.e., patient not attending work, school, etc.) with the “disruption of family routine activities” scale was significantly lower than the alternative scale of “financial burden” (*t* = 2.04, *P* < 0.05).

**Table 2:** Item-scale correlation: convergent and discriminative validity of FBIS (n = 1478)

**Scales**	**Range of correlation coefficients**		**Convergent validity test**		**Discriminative validity test**	
	**Convergent**	**Discriminative**	**No. succeed/**	**Successful**	**No. succeed/**	**Successful**
	**Validity**	**Validity**	**No. tested**	**Ratio (%)**	**No. tested**	**Ratio (%)**
Financial burden	0.61-0.81	0.33-0.67	6/6	100	29/30	96.67
Disruption of family routine activities	0.67-0.81	0.38-0.73	5/5	100	24/25	96.00
Disruption of family leisure	0.80-0.88	0.47-0.74	4/4	100	20/20	100
Disruption of family interactions	0.71-0.86	0.47-0.68	5/5	100	25/25	100
Effect on physical health of others	0.81-0.81	0.39-0.72	2/2	100	10/10	100
Effect on mental health of others	0.78-0.78	0.46-0.71	3/3	100	15/15	100

A principal component analysis and scree plot suggested a model of up to three factors for the FBIS (Table 1). The Kaiser-Meyer-Oklin value was 0.95 (> recommended value of 0.6) and Barlett's Test of Sphericity reached statistical significance (*χ*^*2*^ = 18571.56, *P* < 0.001), supporting the factorability of the correlation matrix. Factor 1 covering the “disruption of family routine activities”, “disruption of family leisure”, and “disruption of family interactions” scales had a large eigenvalue of 13.80 and accounted for 55.21% of the variance. Factor 2 covering the “financial burden” scale explained 9.23% of variance. Factor 3 covering the “effect on physical health of others” and “effect on mental health of others” scales explained 5.71% of the variance.

**Figure 1:**
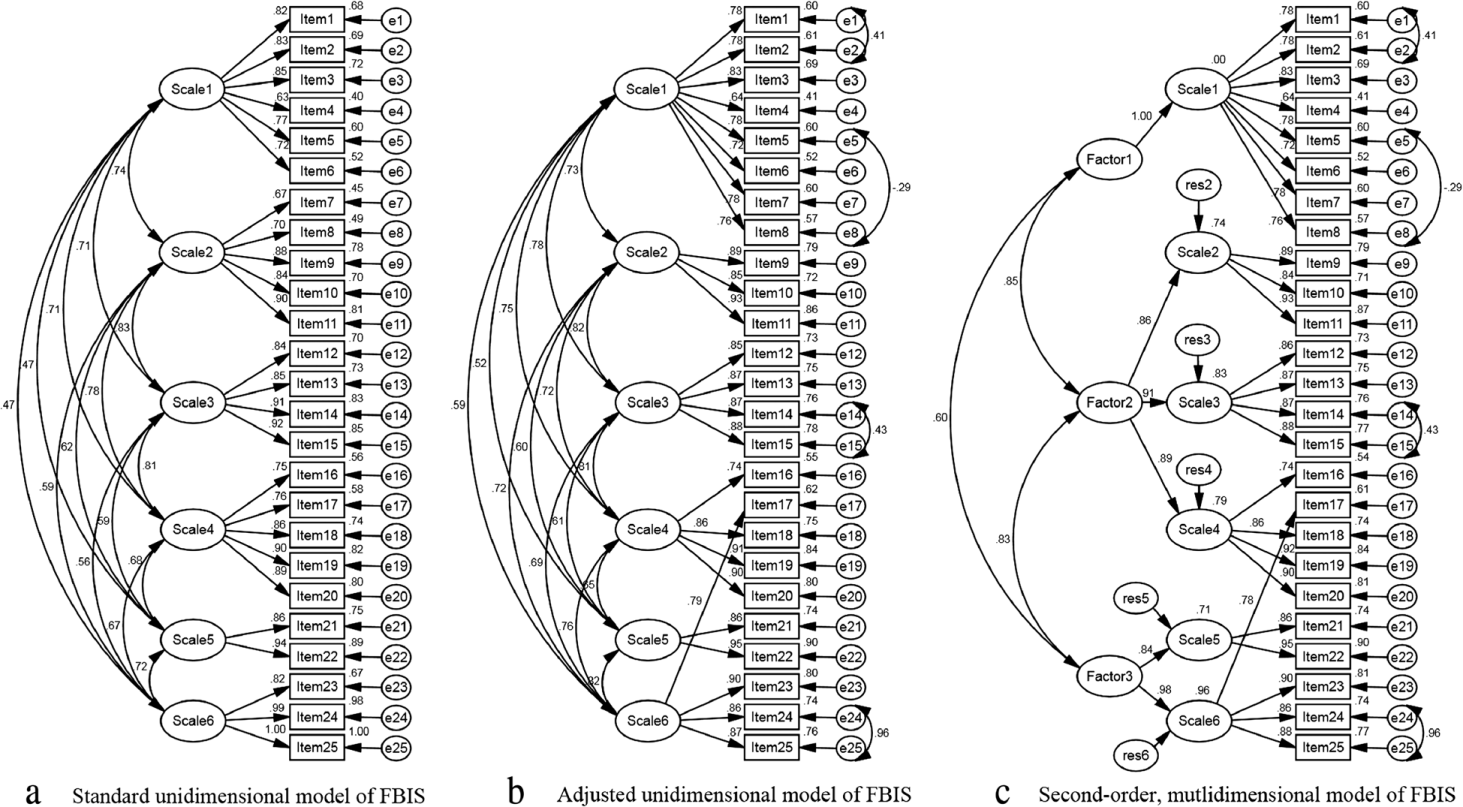
**Structural equation models of confirmatory factor analysis.** Figure 1**a** depicted a standard unidimensional model of FBIS with good fit statistics (NFI = 0.92, CFI = 0.93, RMR = 0.02, RMSEA = 0.08, except GFI = 0.83 and AGFI = 0.79). Figure 1**b** depicted an adjusted unidimensional model with even better fit statistics (GFI = 0.90, NFI = 0.95, CFI = 0.96, RMR = 0.02, RMSEA = 0.06, except AGFI = 0.88). The adjusted model was restructured according to the covariations among items (i.e. item 1 and 2, item 5 and 8, item 14 and 15, item 24 and 25) and results of exploratory factor analysis (i.e. item 7 and 8 were relocated from scale 2 to scale 1, item 17 was relocated from scale 4 to scale 6). Figure 1**c** depicted a second-order mutlidimensional model and its fit statistics were as good as the adjusted unidimensional one.

The confirmatory factor analysis was conducted using structural equation models, and the detailed information is shown in Figure 1.

### Caregiver burden and its determinants

According to the results of the exploratory and confirmatory factor analysis, the range of items of the standard version of FBIS was restructured for the adjusted FBIS. The average score of the burden reported by caregivers of chronic viral hepatitis patients in Shanghai, China on both the standard and the adjusted FBIS was 12.62 ± 10.74. Among the 6 scales, the average score of “financial burden” was 4.81 ± 3.36 on the standard FBIS and 6.29 ± 4.39 on the adjusted FBIS, which constituted the major effect on the family caregiver burden. The average score of “effect on physical health of others” was 0.52 ± 0.91 on both the standard and the adjusted FBIS, which constituted the minor effect (Table 3).

**Table 3:** Means and standard deviations of FBIS for chronic viral hepatitis (n = 1478)

**Scales**	**Standard FBIS**			**Adjusted FBIS**		
	**Range of items**	**Mean**	**Standard deviation**	**Range of items**	**Mean**	**Standard deviation**
Family Burden Interview Schedule	1-25	12.62	10.74	1-25	12.62	10.74
Financial burden	1-6	4.81	3.36	1-8	6.29	4.39
Disruption of family routine activities	7-11	2.68	2.57	9-11	1.20	1.56
Disruption of family leisure	12-15	1.89	2.10	12-15	1.89	2.10
Disruption of family interactions	16-20	2.04	2.38	16,18-20	1.75	1.99
Effect on physical health of others	21-22	0.52	0.91	21-22	0.52	0.91
Effect on mental health of others	23-25	0.68	1.27	17,23-25	0.97	1.64

FBIS: The Family Burden Interview Schedule; Adjusted FBIS was the standard version of FBIS which was restructured according to the results of exploratory and confirmatory factor analysis.

In this study, the total standard FBIS score, which was the same as the adjusted FBIS score, was used as the outcome variable in the two-level random intercept model; it confirmed that the risk factors for burden among the family members with chronic viral hepatitis of patients in Shanghai were hospitalisation (β 1.69, 95%CI 0.48 to 2.90), elevated serum alanine aminotransferase levels (β 1.05, 95%CI 0.15 to 1.95), HCV infection (β 4.49, 95%CI 1.22 to 7.77), and acceptance of the hepatitis B vaccine (β 2.20, 95%CI 0.56 to 3.85), whereas the protective factors which could alleviate the family caregiver burden were no consumption of alcohol (β -2.69, 95%CI −5.19 to −0.19), average monthly costs for patients less than or equal to 100 US dollars (β -2.96, 95%CI −5.83 to −0.09), and good health status of family caregivers (β -9.91, 95%CI −12.76 to −7.05). The estimation of the covariance parameters showed that the residual of the model was 72.43 (P < 0.001), the variance of intercept variable (community) was 31.86 (P < 0.001) and the intra-class correlation was 31.86/(31.86 + 72.43) = 0.31, which supported the existence of a hierarchical structure between the household and the community level. The detailed information is shown in Table 4.

**Table 4:** Determinants of family caregiver burden with two-level random intercept model (n = 1478)

**Characteristic**	**Value**	**β value**	**Std. error**	**t**	***P *** **value**	**95% confidence interval**	
						**Lower bound**	**Upper bound**
**Intercept**		25.52	4.66	5.48	0.00	16.37	34.66
**Patients**							
Gender - no. (%)							
Male	951 (64.34)	-0.75	0.86	-0.87	0.38	-2.44	0.94
Female	527 (35.66)	0^a^	0				
Age - years	51.15(SD 13.28)						
Type of hepatitis - no. (%)							
Hepatitis B	1308 (88.50)	1.45	1.30	1.12	0.26	-1.09	4.00
Hepatitis C	88 (5.95)	4.49	1.67	2.69	0.01	1.22	7.77
Hepatitis B and D	2 (0.14)	3.64	9.08	0.40	0.69	-14.18	21.46
Unidentified	80 (5.41)	0^a^	0				
Clinical diagnosis - no. (%)							
Chronic viral hepatitis	1372 (92.83)	-1.73	2.86	-0.61	0.55	-7.33	3.88
Cirrhosis	95 (6.43)	2.46	3.00	0.82	0.41	-3.43	8.35
Liver cancer	11 (0.75)	0^a^	0				
Antiviral therapy - no. (%)							
Yes	1115 (75.44)	1.14	0.69	1.65	0.10	-0.22	2.50
No	363 (24.56)	0^a^	0				
Hospitalisation or outpatient in the last year - no. (%)							
Hospitalisation	889 (60.15)	1.69	0.62	2.74	0.01	0.48	2.90
Outpatient	589 (39.85)	0^a^	0				
The mean levels of serum alanine aminotransferase in the past 6 months - (IU/L)	222.32 (659.55)	1.05	0.46	2.29	0.02	0.15	1.95
Insurance for medical care - no. (%)							
For urban residents	594 (40.19)	-1.44	1.48	-0.97	0.33	-4.34	1.46
For urban workers	565 (38.23)	-1.02	1.48	-0.69	0.49	-3.93	1.89
New rural cooperative	160 (10.83)	-0.61	1.65	-0.37	0.71	-3.84	2.63
Private expense	113 (7.64)	-1.16	1.70	-0.68	0.50	-4.50	2.18
Commercial insurance	46 (3.11)	0^a^	0				
Alcohol consumption - no. (%)							
No consumption of alcohol	1263 (85.45)	-2.69	1.28	-2.11	0.04	-5.19	-0.19
Less than or equal to 1 time per week	140 (9.47)	-2.03	1.46	-1.39	0.16	-4.88	0.83
More than 1 time per week	75 (5.08)	0^a^	0				
Average monthly costs for patients - no. (%)							
Less than or equal to 100 US dollars	910 (61.57)	-2.96	1.46	-2.02	0.04	-5.83	-0.09
Less than 500 and more than 100 US dollars	515 (34.84)	-0.90	1.50	-0.60	0.55	-3.85	2.04
More than 500 US dollars	53 (3.59)	0^a^	0				
**Family caregiver**							
Gender - no. (%)							
Male	561 (37.96)	-0.78	0.82	-0.95	0.34	-2.39	0.83
Female	917 (62.04)	0^a^	0				
Age - years	51.31(SD 13.66)	-0.06	0.04	-1.59	0.11	-0.13	0.01
Relationship - no. (%)							
Parents	139 (9.40)	1.66	2.01	0.83	0.41	-2.28	5.59
Spouse	1133 (76.66)	0.78	1.07	0.73	0.47	-1.32	2.88
Siblings or children	206 (13.94)	0^a^	0				
Health status - no. (%)							
Healthy	966 (65.36)	-9.91	1.46	-6.81	0.00	-12.76	-7.05
Ordinary	428 (28.96)	-6.65	1.47	-4.51	0.00	-9.54	-3.76
Bad	84 (5.68)	0^a^	0				
Average monthly household income - US dollar	1219.06(SD 2827.33)	5.20e-6	1.45e-5	0.360	0.72	-2.33e-5	3.37e-5
Infected or not - no. (%)							
Yes	199 (13.46)	-0.78	0.84	-0.93	0.35	-2.42	0.86
No	1279 (86.54)	0^a^	0				
Acceptance of the hepatitis B vaccine - no. (%)							
Yes	742 (50.20)	2.20	0.84	2.62	0.01	0.56	3.85
With no answer	503 (34.03)	1.57	0.86	1.82	0.07	-0.12	3.25
No	233 (15.77)	0^a^	0				
Acceptance of regular interviews - no. (%)							
Yes	894 (60.49)	-0.54	0.83	-0.65	0.52	-2.17	1.09
With no answer	315 (21.31)	-1.46	1.09	-1.35	0.18	-3.59	0.67
No	269 (18.20)	0^a^	0				
Nursing time of family caregiver - years	12.56(SD 12.39)	0.02	0.03	0.78	0.44	-0.03	0.07

^a^This parameter is set to zero because it is redundant.

## Discussion

To the best of our knowledge, this research is the first to use FBIS to estimate the caregiver burden in family members of chronic viral hepatitis patients and the first to have satisfactory psychometric properties. Cronbach’s alpha coefficient of FBIS was 0.90 for the overall instrument and 0.88-0.91 for the six correlative scales, which showed an even higher level of reliability than the instrument used in the schizophrenia research [13]. Convergent and discriminative validity with exploratory factor analysis were used to examine the validity of FBIS, suggesting that a limited number of items should be relocated to increase the discriminative ability of the instrument. A second-order multidimensional structure should be attempted with consideration of the covariations among the scales. Further evidence of the confirmatory factor analysis indicated that item 7 (i.e., “patient not attending work, school, etc.”), item 8 (i.e., “patient unable to help in household duties”), and item 17 (i.e., “other members arguing over the patient”) should be relocated from the “disruption of family routine activities” scale to the “ financial burden” scale. Considering the traditional values, family responsibilities and contributions in the Chinese population, not attending work, being unable to help with household duties or causing family conflicts affected routine family activities as well as family income, the distribution of family labour, enthusiasm for work and responsibilities. No item should be removed, which was partly consistent with the results of Chien WT and Norman I [13].

In our study, community-based chronic viral hepatitis patients were recruited, which contributed to estimating the family caregiver burden of inpatients as well as outpatients. With the FBIS measurement, the average score of the burden related to chronic hepatitis in Shanghai was 12.62 ± 10.74 and the financial burden was identified as the major effect. A recent study in Shandong province in China suggested that the annual direct cost for patients with chronic hepatitis B infection, compensated cirrhosis, decompensated cirrhosis or primary liver cancer were 4552 US dollars, 7400.28 US dollars, 6936 US dollars and 10635 US dollars, respectively, which were catastrophic expenditures for the households of the patients [25]. The average scores of “effect on physical and mental health of others” were the lowest in our study, which were not consistent with the actual infection status among the family members of chronic viral hepatitis patients in Shanghai [26,27]. Chinese traditional values, the spirit of dedication, the lack of self-protection, and low self-care awareness among family members might influence the assessments.

To alleviate the errors caused by multistage cluster sampling, the multilevel statistical model (e.g., the two-level random intercept model) was applied to identify the independent variables of the family caregiver burden between the household and the community level [24]. The findings indicated that hospitalisation, elevated serum alanine aminotransferase levels, HCV infection and acceptance of the hepatitis B vaccine were the significant risk factors, whereas no consumption of alcohol, average monthly costs for patients less than or equal to 100 US dollars, and good health status of family caregivers were the protective factors. Hospitalisation was associated with the high cost of laboratory tests and medicines, elevated serum alanine aminotransferase levels typically suggested the severity of liver injury [28], and no consumption of alcohol generally reduced the risk of liver injury [29]. These factors were shown to influence the family burden in this study. Average monthly costs for patients ≤ 100 US dollars suggested a low financial burden in China, and good health status of family caregivers might represent the ability to take care of patients. They were shown to reduce the family burden in the study. Since the 1990s, there have been several reports on the prevalence of HCV infection among HCC or primary liver cancer patients [2]. In China, HBV infection is the major public health problem, whereas the incidence of HCV infection has been increasing in recent years. Considering the generally asymptomatic nature of the disease [30], that there is no available vaccine, weak public awareness, and the marginalisation of patients or at-risk populations, family caregivers of HCV patients might have suffered more emotional pain and discrimination than those of HBV patients in China.

In our study, medical care insurance and average monthly household income had no significant relationship with the family burden. Patients with chronic viral hepatitis generally relied on antiviral therapy to control their viral loads. According to the strict rules on the type and quantity of medical insurance-provided drugs in China, patients or their caregivers must visit hospitals frequently for sufficient drugs which would increase the disease burden. There might be information bias in the average monthly household income item because Chinese tradition opposes revelations concerning information about family wealth. In this study, the most difficult to interpret findings concerned the “side effect” of acceptance of the hepatitis B vaccine. After repeat interviews with the families of several patients, we discovered that in addition to concern about the vaccine quality and price, social discrimination played a vital role in this issue. To avoid discrimination and unfair treatment, many chronic viral hepatitis patients with and their family members chose hidden lives; their colleagues, friends, neighbours, and even relatives were unaware of the health status of the patient [31]. The acceptance of the hepatitis B vaccine or regular community interviews might disturb their private lives and force them to face real situations, which greatly increased the psychological burden of family members. Increased emphasis should be placed on the influencing factors as well as on the quality of community-based services.

Our study has two limitations. First, to explore the determinants among family caregivers of chronic viral hepatitis patients, we focused on the main effect of the first level variables (the individual level) and controlled the hierarchical effect in the database rather than explore the second level variables (the community level) and the relationship between them. We used the two-level random intercept model instead of the two-level random coefficient model in this study. We will explore the significant effects of the community level variables in further work. Second, considering that the hierarchical structure existed in the database, the two-level confirmatory factor analysis might be the best choice. To guarantee the validated and precise estimation parameters and model, with the limitation of the sample size, our research only separately met the requirements of the two-level model or the confirmatory factor analysis.

## Conclusions

The FBIS instrument accurately measures the population-based burden on the family caregivers of chronic viral hepatitis patients, and the financial burden constituted the major effect on the family caregiver burden in Shanghai, China. Targeting intervention toward the conditions associated with chronic viral hepatitis challenges, specific measures, including a standardised antiviral treatment program, health education for patients and their family members, psychological counselling, governmental medical-cost sharing and reduction, and improvement in community-based services, should be implemented.

## Abbreviations

HBV: Hepatitis B virus; HCV: Hepatitis C virus; FBIS: The Family Burden Interview Schedule; GFI: Goodness-of-Fit Index; AGFI: Adjusted Goodness-of-Fit Index; NFI: Normed Fit Index; CFI: Comparative Fit Index; RMR: Root Mean Square Residuals; RMSER: Root Mean Square Error of Approximation.

## Competing interests

None of the authors have any associations that might be deemed a conflict of interest to the publication of this manuscript.

## Authors’ contributions

HR designed the study and directed its implementation. YY assisted with data collection and drafted the manuscript. JYH prepared the Materials and Methods sections of the text. YS assisted with data collection. YHL conducted the literature review and prepared the Discussion section of the text. WM designed the study's analytic strategy, including reliability and validity tests of FBIS and the two-level random intercept model. All authors read and approved the final manuscript.

## Pre-publication history

The pre-publication history for this paper can be accessed here:

http://www.biomedcentral.com/1471-2334/14/82/prepub

## References

[B1] Lavanchy D Worldwide epidemiology of HBV infection, disease burden, and vaccine prevention J Clin Virol 2005 14 Suppl S1 S3 16461208 10.1016/s1386-6532(05)00384-7

[B2] Shepard CW Finelli L Alter MJ Global epidemiology of hepatitis C virus infection Lancet Infect Dis 2005 14 558 567 10.1016/S1473-3099(05)70216-4 16122679

[B3] Nguyen LH Nguyen MH Systematic review: Asian patients with chronic hepatitis C infection Aliment Pharmacol Ther 2013 14 10 921 936 10.1111/apt.12300 23557103

[B4] Tanaka M Katayama F Kato H Tanaka H Wang J Qiao YL Inoue M Hepatitis B and C virus infection and hepatocellular carcinoma in China: a review of epidemiology and control measures J Epidemiol 2011 14 6 401 416 10.2188/jea.JE20100190 22041528 PMC3899457

[B5] Sun Z Ming L Zhu X Lu J Prevention and control of hepatitis B in China J Med Virol 2002 14 3 447 450 10.1002/jmv.10094 12116043

[B6] Huang C Qiu F Guo M Yi Y Shen L Wang F Jia Z Ma J Zhao Y Zhang S Zhang Y Bi S Prevalence and risk factors of hepatitis C among former blood donors in rural China Int J Infect Dis 2012 14 10 e731 e734 10.1016/j.ijid.2012.05.1035 22796320

[B7] Annandale The Sociology of Health and Medicine: a critical introduction 1998 Cambridge: Polity Press

[B8] Barkow J Cosmides L Tooby J The adapted mind: evolutionary psychology and the generation of culture 1992 Oxford: Oxford University Press

[B9] Pai S Kaipur RL The burden on the family of a psychiatric patient: development of an interview schedule Br J Psychiatry 1981 14 332 335 10.1192/bjp.138.4.332 7272637

[B10] Grover S Dutt A Perceived burden and quality of life of caregivers in obsessive-compulsive disorder Psychiatry Clin Neurosci 2011 14 5 416 422 10.1111/j.1440-1819.2011.02240.x 21851450

[B11] Mattoo SK Nebhinani N Kumar BA Basu D Kulhara P Family burden with substance dependence: a study from India Indian J Med Res 2013 14 4 704 711 23703337 PMC3724250

[B12] Martorell A Pereda A Salvador-Carulla L Ochoa S Ayuso-Mateos JL Validation of the Subjective and Objective Family Burden Interview (SOFBI/ECFOS) in primary caregivers to adults with intellectual disabilities living in the community J Intellect Disabil Res 2007 14 Pt 11 892 901 17910541 10.1111/j.1365-2788.2007.00962.x

[B13] Chien WT Norman I The validity and reliability of a Chinese version of the family burden interview schedule Nurs Res 2004 14 5 314 322 10.1097/00006199-200409000-00006 15385868

[B14] Chen H Meng H Lu ZX Prospective study on family burden following traumatic brain injury in children Zhonghua Liu Xing Bing Xue Za Zhi 2006 14 4 307 310 16875532

[B15] Duan QH Xu YC Liang XY Yuan XB Zhao HH Ran P Study on the reliability, validity and sensitivity of a family burden scale used for evaluation on schistosomiasis Zhonghua Liu Xing Bing Xue Za Zhi 2008 14 12 1189 1192 19173961

[B16] Chinese Society of Hepatology, Chinese Medical Association; Chinese Society of Infectious Diseases, Chinese Medical Association Guideline on prevention and treatment of chronic hepatitis B in China (2005) Chin Med J (Engl) 2007 14 24 2159 2173 18167196

[B17] Ghany MG Strader DB Thomas DL Seeff LB American association for the study of liver diseases. Diagnosis, management, and treatment of hepatitis C: an update Hepatology 2009 14 4 1335 1374 10.1002/hep.22759 19330875 PMC7477893

[B18] Hepatotogy Branch, Infectious and Parasitology branch, Chinese Medical Association Guideline of prevention and treatment of hepatitis C Zhonghua Yu Fang Yi Xue Za Zhi 2004 14 3 210 215 15182496

[B19] Nunnally J Bernstein I Psychometric Theory 1994 New York: McGraw-Hill

[B20] Ware JE Gandek B Methods for testing data quality, scaling assumptions, and reliability: The IQ-QLA Project approach. International Quality of Life Assessment J Clin Epidemiol 1998 14 945 952 10.1016/S0895-4356(98)00085-7 9817111

[B21] Jacob C Patricia C Stephen GW Leona SA Applied multiple regression and correlation analysis for the behavioral sciences 2003 3 London: Lawrence Eribaum Associates

[B22] Jolloffe IT Principal Components Analysis 2002 2 New York: Springer

[B23] Byrne BM Structural Equation Modeling with Amos: Basic Concepts, Applications, and Programming 2001 New Jersey: Lawrence Erlbaum Associates

[B24] Goldstein H Multilevel Statistical Models 2011 4 London: Wiley

[B25] Lu J Xu A Wang J Zhang L Song L Li R Zhang S Zhuang G Lu M Direct economic burden of hepatitis B virus related diseases: evidence from Shandong, China BMC Health Serv Res 2013 14 37 10.1186/1472-6963-13-37 23368750 PMC3572417

[B26] Zhang HW Yin JH Li YT Li CZ Ren H Gu CY Wu HY Liang XS Zhang P Zhao JF Tan XJ Lu W Schaefer S Cao GW Risk factors for acute hepatitis B and its progression to chronic hepatitis in Shanghai, China Gut 2008 14 12 1713 1720 10.1136/gut.2008.157149 18755887 PMC2582333

[B27] Li J Liu JY Hu JY Li YT Study on the status of HBV infection and its determinants among family members with HBV infected persons in Shanghai Zhonghua Liu Xing Bing Xue Za Zhi 2013 14 3 205 209 23759221

[B28] Tsang PS Trinh H Garcia RT Phan JT Ha NB Nguyen H Nguyen K Keeffe EB Nguyen MH Significant prevalence of histologic disease in patients with chronic hepatitis B and mildly elevated serum alanine aminotransferase levels Clin Gastroenterol Hepatol 2008 14 5 569 574 10.1016/j.cgh.2008.02.037 18455697

[B29] Lee M Kowdley KV Alcohol’s effect on other chronic liver diseases Clin Liver Dis 2012 14 4 827 837 10.1016/j.cld.2012.08.010 23101984

[B30] Hajarizadeh B Grebely J Dore GJ Epidemiology and natural history of HCV infection Nat Rev Gastroenterol Hepatol 2013 [Epub ahead of print] 10.1038/nrgastro.2013.107 23817321

[B31] Na L Na B A revolutionary road: an analysis of persons living with hepatitis B in China J Health Commun 2013 14 1 71 91 10.1080/10810730.2012.688449 23171308

